# Union Meeting of the Seventh and Eighth District Dental Societies

**Published:** 1884-03

**Authors:** 


### UNION MEETING OF THE SEVENTH AND EIGHTH DISTRICT DENTAL SOCIETIES.

At a joint convention of the Seventh and Eighth District Dental Societies of the State of New York, held in Buffalo, October 30th and 31st, 1883, Dr. C. T. Howard, of Rochester, introduced the following preamble and resolution, which, after a full and free discussion, was laid upon the table indefinitely.

"In view of the progress made by the dental profession in the State of New York, and the better control of its highest interests obtained through continued working of wise and prudent legislation in its behalf, and the increasing belief in a thorough course of education as the only proper channel by which new members should in future be admitted to its ranks; be it

"*Resolved,* That the Seventh and Eighth District Dental Societies of the State of New York, assembled in Buffalo in joint convention, recommend the abolishing of the power of the State Board of Censors and the Dental Society of the State of New York, to grant diplomas, licenses, or certificates of admission to the private practice of dentistry."

*Dr. Barrett.*—I second the resolution, for the purpose of bringing it before the convention for discussion. The matter will undoubtedly come up at the next meeting of the State Society, and I think a thorough discussion of the subject should be had previous to that time.

*Dr. Line.*—I shall vote against the resolution, as it seems to be in the interest of the "National Board of Dental Examiners." I am most heartily opposed to that Board. There are good men in it, and there are bad men in it; but it will take a long time to undo the bad work done at Niagara Falls. I am against all such methods as were there adopted, or any resolution which in any manner seems to be in the interest of that Board.

*Dr. C. T. Howard.*—I disclaim any intention of presenting this resolution in the interest of the "National Board of Dental Examiners." I present it in the interest of no Board whatever, but in the interest of dental progress and higher education. I feel that something should be done to take away from our State Board of Censors the power to grant diplomas and certificates of qualification to practice dentistry. A college course should be required of all men entering the profession in future.

*Dr. Freeman.*—I desire to say a word against the resolution. It has no place in this convention. This is not an official body, and cannot act upon such matters. I do not think the State Society would be offended if such a resolution should come to it from this body, yet it could have no weight. It should come, if at all, from the District Societies at their annual meeting in the spring; then the State Society would be bound to notice it.

*Dr. Ellis.*—It is very evident that there will be a minority report on this subject. But I would say to you, gentlemen, that it has got to come to this. It is a question of progress, and not of preference, and it will not be long before the profession will demand the abolition of this Board. Its effect is to cheapen dentistry by admitting incompetent men to the profession. There is no doubt that a man can pass a State Board at a less expenditure of time and money than a regular dental college course requires, and this is why I say the tendency is to cheapen dentistry.

*Dr. Lenox.*—It was just so with me. It was an open question for a long time whether I would read up, go before the Board and get a diploma, or close my office, go to a college, and so enter the profession in that way. I chose the latter course, and should recommend it to any young man studying dentistry.

*Dr. Butler.*—I am opposed to the resolution. I am for standing by our "State Board of Censors." It has done a work in this State that no college could do, and it has not yet finished that work. No educational institution can do the work of that Board, and why any gentleman should desire to cut it off in the midst of its usefulness, I cannot understand. In matters of education it is first and foremost in all that relates to dental progress. Its requirements of the applicant are far higher than any dental college in the country. I believe in colleges. I am not opposed to them in any sense. Indeed, I would urge upon any young man the desirability of finishing his studies with a course at a first-class dental college. The mistake is, I think, in comparing our Board of Censors to an educational institution. Colleges are to educate and prepare young men for the profession; the Board to determine whether the applicant is thus qualified. That is all; and while it is a fact that colleges do sometimes graduate men unfit for the practice of dentistry, I ask you, gentlemen, to point to one M. D. S. who is unworthy of the degree. These are facts upon the side of our Board of Censors, and they are some of the reasons why I am opposed to the resolution.

*Dr. Burkhart.*—I believe in our Board, and shall stand by it in voting against the resolution. New York State stands in the front rank in dentistry, as in nearly everything else, and I believe that no little credit is due this Board for the fact; therefore I vote to sustain and encourage it in the work yet to be done.

*Dr. French.*—Read the law under which the Board was organized and governed, which was clearly in harmony with all our dental colleges. There was no antagonism between the Board and the colleges, in the speaker's judgment.

On motion of Dr. Ellis, seconded by Dr. C. T. Howard, the resolution was laid upon the table by a unanimous vote of the convention.

